# Tongue Behavior in Anterior Open Bite—A Narrative Review

**DOI:** 10.3390/diagnostics15060724

**Published:** 2025-03-14

**Authors:** Olimpia Bunta, Ioana Filip, Cristina Garba, Ioana-Maria Colceriu-Simon, Cristian Olteanu, Dana Festila, Mircea Ghergie

**Affiliations:** Orthodontics Department, Faculty of Dental Medicine, Iuliu Hațieganu University of Medicine and Pharmacy, 400012 Cluj-Napoca, Romania

**Keywords:** anterior open bite, tongue thrust, tongue interposition, tongue influence, swallowing disorder

## Abstract

**Background/Objectives**: Multiple factors may contribute to the development of open bite malocclusion, including genetics and environmental factors. Anterior open bite is usually related to the interposition of the tongue between the dental arches during swallowing or at rest. But how important is the role of the tongue in the development of anterior open bite? And how does anterior open bite malocclusion influence tongue behavior? With this study, we would like to offer a better understanding on the importance of tongue function in the context of this malocclusion. **Methods**: In this narrative review, a comprehensive electronic search was conducted via PubMed, Google Scholar, and ScienceDirect. The inclusion criteria were original research articles published between 2014 and 2024 with full text access. The exclusion criteria were articles older than 10 years and articles with restricted access or without full text access. **Results**: Out of the initial 1231 articles, 9 articles were found to be eligible for the present study. The tongue is a part of the neutral zone of the oral cavity, together with the lips and cheek musculature, forming a corridor of equilibrium. If this neuromuscular balance is altered, the teeth will move out of the neutral zone, and various malocclusions may develop. Patients with anterior open bite experience difficulties in closing the anterior portion of the oral cavity during swallowing; therefore, adaptative changes occur in an attempt to compensate by changing the dynamics of the tongue. **Conclusions**: The cause–effect relationship between tongue malfunction and anterior open bite remains controversial. This review article mentions the possible role of the tongue in anterior open bite etiology, but evidence is still needed on this subject, as it is yet unclear whether the influence of the tongue is a primary cause, an influencing factor, or just a consequence of an already-existing vertical occlusal pathology.

## 1. Introduction

Open bite malocclusion is a vertical discrepancy characterized by a lack of contact between the upper and the lower teeth [1,2]. Depending on the location of the teeth involved, open bite can be classified as anterior open bite (usually the absence of vertical contact between the maxillary and mandibular teeth from canine to canine) or as posterior open bite (involving the teeth located distal to the canines). Anterior open bite (AOB) is more common than posterior open bite, with a prevalence of 16.52% in children and adolescents aged 2–16 years [3]. Similar results were found in other studies, where anterior open bite was discovered in 17–18% of children with mixed dentition [4,5,6,7]. Meng-Zhao Deng et al. found that in the American population aged 8–17, the prevalence of anterior open bite was 3.5%, with 3–4 times higher values in the East African population compared to those of Caucasians [8]. In a study published by Paula Gonzalez et al., a prevalence of 2–4.7% in Caucasians is mentioned [9]. It has been found that the prevalence of anterior open bite is higher in deciduous and early mixed dentition (11%), and it is less common in late mixed dentition (4.2–6.2%). In permanent dentition, a percentage of 2.5–8.7% has been found [9].

This malocclusion has two clinical forms: skeletal open bite, which is associated with the excessive vertical growth of the dentoalveolar complex in the posterior maxillary region, and dental open bite, which is related to the reduced vertical height of the dentoalveolar complex in the incisor region [10,11,12,13]. Patients with skeletal open bite exhibit an increased lower anterior face height, a short posterior face height, an increased mandibular plane angle, an increased gonial angle, an increased height of the maxillary molar dentoalveolar complex [14,15], a hyperdivergent facial type with a clockwise mandibular rotation, a retrognathic mandibular position, and/or counterclockwise maxillary rotation [16]. On the other hand, patients with dental open bite present a normal craniofacial pattern, with a balanced lower anterior facial height [10] but with protruded and under-erupted frontal teeth [10,16].

There are data in the literature about a third clinical type of open bite, functional open bite [10]. Due to the intricate nature of the open bite malocclusion etiology and symptomatology, differentiating between the clinical forms of this anomaly is difficult. Nevertheless, the literature suggests that functional open bite is associated with head and neck muscle-related behaviors within the oral cavity: breathing, speech, mastication, and deglutition [16].

Multiple factors may contribute to the development of this malocclusion, including genetic and environmental factors, but bad habits such as thumb sucking, tongue thrusting, and prolonged pacifier use are also involved [10]. Many authors have studied the role of genetic and epigenetic mechanisms in anterior open bite, as genetic factors alone are not the sole influencer in malocclusion development. Epigenetic factors, histone modification, DNA methylation, and micro-ARN regulation have been found to modify patterns of genetic expression, impacting growth, the development of maxillary and mandibular bones, and tooth development [10]. Some of the genes involved are FGRR2—linked to Crouzon and Apert syndromes; TCOF1—related to Treacher Collins syndrome; FAM83H; ENAM; AMGX; PAX5; PTPN11; and SOS1 [16,17,18].

Variations in dental eruption or alveolar growth, disproportionate neuromuscular growth, tongue malfunction, and/or bad oral habits are determined by environmental factors [19,20]. The mechanism involved in the development of anterior open bite due to prolonged thumb sucking, pacifier use, and tongue thrusting during childhood is explained by the mechanical forces applied on the teeth, thereby influencing alignment and determining the development of anterior open bite [16,21,22,23,24,25,26,27,28]. Oral breathing, especially if chronic, can also influence facial growth and be a risk factor in open bite etiology. Lifestyle, including diet, medication, different general pathologies, and stress as environmental factors, might also be responsible in the etiology of open bite [16,29,30].

Macroglossia with pathological associations, including amyloidosis, Beckwith–Wiedeman syndrome, and acromegaly, might also induce a tongue-thrusting habit [16].

Open bite is usually related to the interposition of the tongue between the dental arches during swallowing or at rest [19]. This positioning of the tongue can be found in the literature under multiple names: tongue thrusting, deviant deglutition, perverted swallowing, abnormal swallowing, reversed swallowing, and oral myofunctional disorder.

There is a debate in the literature regarding the role of the tongue in the development of open bite, whether it is a cause or an effect of this malocclusion. 

With this study, we would like to offer a better understanding of the behavior and dynamics of the tongue in the anterior open bite clinical picture.

## 2. Materials and Methods

A narrative review of the literature was conducted to explore the correlation between tongue position and function, and anterior open bite etiology.

Information Sources and Search Strategy: A comprehensive electronic search was conducted via: PubMed, Google Scholar, and ScienceDirect, as summarized in Table 1. The search used keywords or terms in English as follows: “anterior open bite”, “tongue thrust”, “tongue interposition”, “tongue influence”, “tongue position”, and “swallowing disorder”. According to the research objective, the inclusion criteria were articles published between 2014 and 2024 with full text access. The exclusion criteria were articles older than 10 years and articles with restricted access. The articles were selected based on their context and legitimacy level, and those that matched the criteria for inclusion were considered relevant to the study, while those that did not were excluded.

Selection and data collection process: JabRef Bibliography Management v.5 was used to automatically deduplicate references. All authors participated in the initial data collection and also in the cross-validation of the findings in order to ensure accuracy. Academic debate helped us to reach a consensus regarding the inclusion and exclusion criteria. After the initial search results, automatic deduplication, and manual selection, the remaining papers were carefully examined one by one. Ultimately, 35 articles were selected for inclusion in this study.

Given the nature of a literature review, no ethical approval was required.

## 3. Results

Even though 1231 articles were found in the first database search, the final collection of 9 articles that were judged suitable for inclusion in this narrative review paper were obtained by applying the inclusion and exclusion criteria. To improve clarity and structure in presenting our results, the studies relevant to our investigation of the role of the tongue in anterior open bite etiology and symptomatology are summarized in Table 2.

### 3.1. Anatomical Characteristics of the Tongue

The tongue is a muscular organ located in the functional space of the mouth, formed by striated muscle tissue [37]. As presented by the Federative International Programme for Anatomical Terminology in their 2019 Terminologia Anatomica, the tongue consists of a set of eight extrinsic or intrinsic muscles—genioglossus, hypoglossus, styloglossus, palatoglossus, superior and inferior longitudinal lingual, transversus linguale, and verticalis linguale [38,39]. The intrinsic muscles of the tongue originate and insert within the tongue organ [40], determining its shape [41,42,43], whereas the extrinsic muscles originate on bones outside the oral cavity, then enter the tongue and interdigitate with other categories of muscles, ultimately inserting themselves within the tongue [40]. The extrinsic action of these muscles determines the positioning of the tongue [41,42,43].

In terms of action, the muscles of the tongue can be divided into two categories: protruders and retruders. The protruder muscles are responsible for the forward extension of the tongue, and the retruder muscles are responsible for the return of the tongue inside the oral cavity. The genioglossus muscle is commonly grouped together with the protruder muscles, but its anterior portion has an actual lowering effect on the tip of the tongue, therefore determining a more retruding action. This fact determines the classification of the muscle action to be related with other aspects, like volume as well as its static anatomy [40].

There is little information in the literature on the relationship between function and tongue anatomy and structure. The reason for this is that in anatomy atlases and dissections on cadavers, only tongue muscles and fibers can be assessed objectively, as their interdigitation makes it difficult or even impossible to dissect out a single muscle in its entirety, unlike other muscles in the human body. Due to this fact, their summative action is very challenging [40].

In order to have a better understanding of the action and overall function of the tongue muscle complex, we must take into account the fact that a muscle is not a neuromuscular unit in itself [39]. As Chanaud et al. described it in 1991, and was then cited by Wrench in 2024, compartmentalization of muscles with distributed origins and insertions might include different biomechanical effects due to the different insertions and/or origins, even if they have similar histological and chemical composition of the fibers [39,44].

### 3.2. Functions of the Tongue

The tongue offers airway protection against spillage and maintains tonic muscle contraction in order to keep the airway open. It also exhibits constant forces on the dental arches and their support structures [8,43,44,45,46]. The tongue is part of the neutral zone together with the lips and cheek musculature, forming a corridor of equilibrium in which the extraoral and intraoral forces must be equal, in order to offer optimal conditions for the teeth to erupt in a normal occlusion. If this neuromuscular balance is altered, the teeth will be out of the neutral zone, and various malocclusions may develop [47].

The tongue actively participates in different functions such as chewing, swallowing, phonetics, and breathing [8,37,48,49,50,51]. During mastication, the tongue movement plays an important role in the formation and transfer of the food bolus towards the esophagus [31]. The muscular organ guards the airway from leakage and maintains tonic muscle contraction to keep it patent. However, it also exerts large sustained and sudden stress on teeth and supporting tissues. Measuring the tongue’s morphology and function can be difficult due to its deep position in the oral cavity and lack of accessibility for many devices. Over the past 30 years, ultrasound imaging has been shown to accurately assess tongue shape and mobility [8].

The coordination between the tongue, palate, teeth, lips, alveolus, and other vocal organs contributes to the development of soundwaves and formation of words [52]. Studies have shown that 80% of the pronunciation of some letters is formed in the anterior part of the mouth. Anterior open bite is the most frequent anomaly associated with speech disorders. The pronunciation of different phonemes such as s, z, th, d, l, f, and v is altered because of the excessive propulsion of the tongue, which is placed in a lower position than in patients without open bite [4,32,33,53].

Normal breathing takes place through the nose when the tongue is at rest. Although physiological breathing plays an active role in the development of a harmonious craniofacial structure, when external factors change its mechanism, its impact on skull development may lead to changes in both function and skeleton. There are three forms of breathing: oral, mixed (oro-nasal), and nasal. Oral breathing is a parafunctional habit in which the air travels through the mouth instead of the nose resulting in an altered positioning of the tongue, head, and jaw. These altered postural changes affect the growth by increasing the anterior face height, rotating the mandible clockwise and opening the bite anteriorly and increasing the overjet [52,53,54,55,56]. A narrow maxillary dental arch results from irregularities in the ramus’s vertical growth as well as greater pressure from stretched cheek muscles [57]. Moreover, the position and movement of the mandible are linked to lingual muscle activity.

The function and position of the tongue are critical to the physiological swallowing process. In children with deciduous and mixed dentition, atypical or improper lingual positions have been associated with facial abnormalities. Often known as tongue tie, ankyloglossia is a congenital oral anomaly that affects swallowing and the stomatognathic system’s growth and development. Children that have an improper swallowing pattern may not touch the front of the palate with their tongue tip [52,53,54,55,56,58,59].

### 3.3. Types of Deglutition

There are two main types of deglutition or swallowing: visceral (infantile) swallowing and somatic (mature) swallowing. Visceral swallowing occurs with a forward movement of the tongue against the lingual surfaces of the anterior teeth or between upper and lower dental arches, and is considered physiological during infancy, during the transition between the temporary and permanent dentitions [19] or until the age of four [58], or six, for some [19,58].

Somatic swallowing involves a cranial movement of the tongue with its tip pressing the incisive papilla, with no lip and cheek activity during the swallowing act [60]. The transition from visceral to somatic deglutition has to occur after the age of four [58] and is usually present in a mixed form during this stage [19]. If this phenomenon does not occur, atypical deglutition might develop, as this is considered the key factor for anterior open bite [58,59,60,61,62,63,64,65,66,67,68].

The shift between the two types of deglutition occurs gradually over a 12–15-month period, along with the dental eruption [55,65]. Considering the etiology of atypical swallowing, two different categories are described in the literature: primary and secondary atypical deglutition [65]. Primary atypical swallowing is mainly associated with parental over-nursing, juvenile behavior, and mood disorders, with a strong psychological impact related to external stressful events. Secondary atypical swallowing is associated with physical factors, described as oral habits (prolonged pacifier user, thumb sucking, bruxism), abnormal tongue posture, extended artificial breastfeeding and weaning, anatomical aspects such as short frenulum, or genetic factors such as the morphology of palatine vault and respiratory tract [65]. Atypical deglutition can be simple or complex, based on the type of tongue thrust and the amount of contraction of orofacial muscles, such as facial, labial, and mental muscles [65]. According to the literature, atypical swallowing is associated with numerous malocclusions, both skeletal (open bite, posterior cross-bite, maxillary protrusion, mandibular retrognathism) and dental (increased overjet and decreased overbite, maxillary incisor protrusion, spacing problems) [59,65].

The main differences between the two types of swallowing are summarized in Table 3.

Patients with anterior open bite experience difficulties in closing the anterior portion of the oral cavity during deglutition; therefore, the tongue and other oral structures try to compensate by changing the tongue dynamics [31,34,69,70]. Gonzalez et al. found that in anterior open bite, 28.8% of patients had tongue contact on the palatal surface of the incisors compared to 13.6% in normal vertical overbite patients; 25.8% of subjects with anterior open bite had contact at the gingival margin versus 28.8% in individuals with no open bite; and only 22% of open bite patients had contact on the palatal rugae versus 53% of patients with normal vertical overbite, during swallowing [9]. When evaluating swallowing tongue contacts in relation to age, other authors found that in the group aged 8–12 years old, tongue thrusting was more frequent than in the 13–16-years-old group [35]. Sayahpour et al. found that patients with visceral deglutition also have lip incompetence in most of the cases and develop a pathological resting position of the tongue [36].

### 3.4. Evaluation of Tongue Pressure in Anterior Open Bite Patients

It is difficult to evaluate the function of the tongue given its inaccessible location. There are quantitative and qualitative means of evaluating the strength of the tongue. The most frequently used method is the qualitative assessment, but it is subjective and dependent on clinician experience [37]. Quantitative methods presented in the literature include cineradiography, electromyography, electropalatographic examination, cinemagnetic resonance, video fluoroscopy, and Payne technique [9]. The quantitative methods are considered more reliable when assessing tongue dynamics. In a study by Kurihara et al. investigating tongue kinetics during deglutition in open bite patients, a quantitative method with a sensor sheet system was used. They discovered that tongue pressure in anterior open bite was weaker from the mid-median to the posterior-median part compared to healthy individuals [31]. The explanation for this might be the abnormal position of the tip of the tongue in anterior open bite patients, which is placed anteriorly between the upper and lower arches, thus preventing strong contact between the tip of the tongue and the anterior-median part of the palate [31]. Another study conducted by Meng-Zhao Deng et al. concluded that during swallowing, patients with anterior open bite showed a greater range and speed of motion, as well as a wider zone of consistent movement of different tongue segments, meaning that in anterior open bite individuals, the tongue exhibits less regional coordination and a decreased fine motor control [8].

### 3.5. Speech Disorders and Anterior Open Bite

In recent years, research studies have focused on the relationship between phonation problems and anterior open bite. Given that AOB malocclusion is most commonly associated with speech difficulties, tongue function changes have received significant attention in this context. The causality relationship between the two deserves further exploration, as it is unknown if AOB leads to phonetic adjustments or phonetic changes cause AOB [33]. Studies in the literature have pointed to the association of a protruded tongue position during swallowing, in contact with the palatal surface of the incisors, and tongue thrust with dental articulation issues [35]. The protruded tongue position during swallowing and tongue positioning during speech must also be associated with the tongue’s position at rest. Kravanja et al. showed that 81.3% of the AOB patients investigated in their study presented an improper tongue posture on the mouth floor [32].

As the study conducted by Ruiz Gutierez et al. shows, the association of the determination of the altered AOB occlusal pattern and the possibility of AOB occurrence in patients with alterations in tongue position during speech, lisping in particular, caused by tongue interposition and thrust, was found to be 12 to 50 times higher [35].

Ocampo-Parra et al. showed that the lack of an articulation point of the phonemes d/t/s/ch forces the tongue to modify its position, protruding between the dental arches and causing interdental tongue thrust, as their study highlighted a high percentage of dyslalia in AOB patients [33]. Sigmatism and rhotacism were also found to be very frequent articulation disorders in AOB patients [32]. Also implicated in the distortion of the phonemes might be the presence of a pulling or short lingual frenulum, but further studies are needed [33].

## 4. Discussion

This review aimed to explore the role of the tongue in the development of anterior open bite. In most of the articles that have been collected, it was discovered that the two are related.

Anterior open bite is considered a challenging malocclusion among orthodontists due to its multifactorial etiology, complex treatment management, and high relapse rate. The implication of the tongue in the development of anterior open bite has been studied for many years in the orthodontic literature, without a special focus on their cause–effect relationship. As demonstrated in the literature, patients with anterior open bite exhibit a forward position of the tongue, resting between the maxillary and mandibular dentition [9,71,72,73,74]. Ruiz Gutierrez et al. concluded in their study that the position and function of the tongue are correlated with modifications such as interposition and tongue thrusting during deglutition and pronunciation in patients presenting anterior open bite [35]. On one hand, this forward positioning of the tongue at rest or during deglutition, exerting constant pressure on oral structures, is considered to be the etiological factor of anterior open bite [10,16,35]. Usually, patients with anterior open bite also present weak perioral musculature; thus, extraoral forces applied by the lips are lower than forces generated by the tongue, producing maxillary incisor proclination and enhancing anterior open bite development [35,73,74,75].

On the other hand, tongue protrusion during deglutition is considered by many authors to be a compensatory mechanism in order to achieve anterior seal in individuals with anterior open bite [8,9,47]. In order to meet the functional demands caused by the dental and craniofacial features, the neuromuscular system has to readapt in order to perform proper deglutition, so the tongue thrusting habit occurs [9,31]. Following this idea, studies have shown that after orthognathic surgery in patients with open bite, tongue kinetics adapted to the new oral environment [31]. In their study, Cenzato et al. address the problem of the cause versus effect regarding the role of the tongue in the case of anterior open bite. They say that the most popular opinion is that the infantile deglutition is caused by the open bite. There is a belief that the pressing duration of the tongue on the anterior teeth during deglutition is too short to have an influence on the eruption of these teeth and on the development of the anterior open bite. For the above-mentioned authors, the incorrect deglutition is caused by the tongue, which is pushed forward during swallowing [19]. Another hypothesis presented in the literature is that, in fact, only the forward lingual resting position can cause anterior open bite because it blocks the vertical eruption of anterior teeth [9]. 

According to Kurihara et al., individuals with anterior open bite and tongue thrusting have a different swallowing pattern than people with a normal overbite [31]. When assessing the tongue behavior during swallowing, a study conducted by Gonzalez et al. presented that the majority of patients with anterior open bite placed the tip of the tongue on the palatal surface of incisors, whereas patients with normal vertical overbite presented tongue contact on the palatine rugae [9]. Deng et al. found that in patients with anterior open bite, the tongue moves faster and more frequently during deglutition, and the thickness of the tongue during chewing is mostly related to skeletal variables than to other functional features of the tongue [8]. Age appears to influence visceral deglutition among children with anterior open bite. Tongue thrust was present more frequently in the group age of 8–12 compared to the 13–16 age group [35]. When assessing visceral swallowing in relation to gender, a study by Debora do Canto Assaf et al., published in 2021, concluded that male gender represented a protective factor for abnormal tongue position [76]. 

Tongue posture during rest was found to be crucial for the development of AOB [32], but tongue-altered positions during functions (swallowing, speech) are also considered to be strongly implicated in AOB etiology.

In order to establish a causality of the association of two or more factors, the literature suggests some formulas, such as the Bradford Hill criteria (nine viewpoints) for causation [77] and/or the John Stuart Mill classical formulation (three viewpoints) [78]. As even the three viewpoints of the Mill formulation (temporal precedence, covariance, and disqualification of alternative explanations) fail to be addressed by the studies found in the literature, an exact causal relationship between tongue and anterior open bite is impossible to be determined. Even though there are a lot of studies on the anterior open bite topic, the specific causative relationship of tongue interference as an etiological factor has yet to be discovered [78].

## 5. Conclusions

After reviewing the literature, the conclusion can be drawn that the position of the tongue is of crucial importance in maintaining occlusal harmony and good dental alignment during the developmental period of the patient. Altered tongue posture, as well as tongue thrusting, can disrupt the balance of forces inside the oral cavity. Patients with open bite malocclusion present different tongue kinetics compared to healthy individuals. The cause–effect relationship between tongue malfunction and open bite remains controversial. The tongue is incriminated both as a risk factor in the etiology of anterior open bite and an adaptative factor in situations in which anterior vertical opening emerges, therefore maintaining and/or amplifying the preexisting pathological condition. To keep a correct dental occlusion, the tongue position should be kept in balance while swallowing but also at rest. Therefore, both position and function of the tongue should be among the first aspects that are checked by the clinicians when consulting an anterior open bite patient.

## Figures and Tables

**Table 1:** Search strategy summary.

Items	Specification
Databases searched	PubMed, Google Scholar and Science Direct.
Search terms used	“anterior open bite”, “tongue thrust”, “tongue interposition”, “tongue influence”, “tongue position”, “swallowing disorder”.
Timeframe	2014–2024.
Article type	Original articles.
Text availability	Full text.
Inclusion criteria	English language only, articles published after 2014, original articles.
Exclusion criteria	Articles not in English language; full text unavailable; editorials, reviews, case reports, opinions, correspondences; articles published prior to 2014.

**Table 2:** Summary of the studies that address the behavior and role of the tongue in anterior open bite malocclusion.

Article Type	Author, Year	Subject/Objectives	Result/Conclusion
Original article	Kasparaviciene et al., 2014 [7]	Assess the relationship of different occlusal traits with oral habits.	Non-nutritive sucking habits and tongue thrust swallowing are significant risk factors for the development of anterior open bite and posterior crossbite in preschool children.
Original article	Deng-Zhao et al., 2019 [8]	Tongue morphology and functional motion, craniofacial skeletal pattern and upper airway space, and speech function in patients with and without anterior open bite.	The tongue moves farther and faster during chewing and swallowing in anterior open bite patients.
Original article	Gonzalez et al., 2019 [9]	Tongue position during deglutition in anterior open bite and normal vertical overbite using a fluorescein technique	AOB group presented a higher prevalence of modified tongue position.
Original article	Kurihara et al., 2019 [31]	The effect of tongue thrusting on tongue pressure production during swallowing in patients with anterior open bite.	Patients with AOB and tongue thrusting in swallowing showed diversity of tongue pressure waveforms and noticeably weaker tongue pressures from mid-median to posterior-median regions than healthy individuals.
Original article	Kravanja et al., 2018 [32]	Associations between the improper tongue posture and articulation disorders in AOB.	Improper tongue posture with higher odds ratio for the presence of AOB.
Original article	Ocampo-Parra et al., 2015 [33]	Types of dyslalia and differences according to the magnitude of AOB.	Phonation alterations are very common in AOB, but further investigation required to elucidate whether AOB leads to phonetic changes or phonetic changes cause AOB.
Original article	MacAvoy et al., 2016 [34]	Determination of the effects of acute change in occlusal vertical dimension on intraoral pressure swallow patterns and perioral electromyographic activity during swallowing.	The adaptive response and the waveform similarities associated with OVD variation support the existence of a central control mechanism for swallowing.
Original article	Ruiz Gutierrez et al., 2021 [35]	Relationship between AOB and tongue position during swallowing and phonation.	The possibility of developing AOB is up to 50 times higher in individuals with phonation alterations caused by tongue interposition and thrust. Also, the possibility of AOB to determine swallowing modifications was 3.3 times higher.
Original article	Sayahpour et al., 2024 [36]	To investigate the association between the visceral swallowing pattern (VSP) and various factors.	Significant correlation of VPS with lip incompetency, pathological resting position of the tongue and AOB and increased anterior overjet.

**Table 3:** Types of deglutition.

	Infantile/Visceral Swallowing	Mature/Somatic Swallowing
Age	Infancy, before 4–6 years of age	After 4–6 years of age.
Dentition	Before or during temporary dentition.	Permanent dentition.
Main characteristic	Forward movement of the tongue against the lingual surfaces of the anterior teeth/between the upper and lower incisors.	Tip of tongue pressing the incisive papilla.
Cephalometric modifications	Lower position of the hyoid bone; reduced pharyngeal airspace.	Normal distance between the hyoid bone and the maxillary plane.
Associated pathologies	Frequent association with temporomandibular disorders; body posture modifications.	Temporomandibular disorders if associated with other malfunctions/bad habits/malocclusion.
Treatment options	Orthodontic approach + myofunctional therapy.	Orthodontic approach.

## Data Availability

No new data were created or analyzed in this study.
